# Advanced Materials for Biomedical Applications, Editorial Article

**DOI:** 10.3390/ma17153692

**Published:** 2024-07-26

**Authors:** René D. Peralta-Rodríguez, Esmeralda Mendoza-Mendoza, Ioannis L. Liakos

**Affiliations:** 1 Centro de Investigación en Química Aplicada, Blvd. Enrique Reyna No. 140, Col. San José de los Cerritos, Saltillo 25294, Mexico; 2 Investigadoras e Investigadores por México-CONAHCYT, Ciudad de México 03940, Mexico; 3 Microscopia de Alta Resolución, Centro de Investigación en Ciencias de la Salud y Biomedicina (CICSAB), Universidad Autónoma de San Luis Potosí, San Luis Potosí 03940, Mexico; 4 Centro de Investigación y Estudios de Posgrado, Facultad de Ciencias Químicas, Universidad Autónoma de San Luis Potosí, San Luis Potosí 78210, Mexico; 5 Institute of Nanoscience and Nanotechnology, National Centre for Scientific Research Demokritos, Patr. Gregoriou E & 27 Neapoleos Street, 15341 Agia Paraskevi, Greece

Advanced materials (AMs) encompass materials that feature improved properties compared to common counterparts. Notably, they can be homogeneous or heterogeneous structures at the molecular level. An important feature of AMs is their size, as measured by the nanometric and the micrometric scales (1 × 10^−9^ to 1 × 10^−6^ m). Further, AMs are characterized by their improved mechanical, chemical, and physical properties, including their high strength, durability, flexibility, and conductivity [1] compared to conventional materials. AMs are not necessarily new; in fact, they can be traced to 3000 B.C.E., when the addition of other metals to copper, mainly tin, produced a material that had superior properties than copper alone, creating the advanced material of bronze. The Egyptians are credited with being the first civilization to produce gold alloys. The development of AMs, mainly alloys (particularly iron alloys) continued through the Roman Era (pozzolanic cement, concrete), the Industrial Revolution (chromium–iron alloys, stainless steel), the Post-Industrial Revolution (improved stainless steel), and up to today, the Technology Age (silicon microchip, new alloys, nanotechnology, polymers, etc.). In this age of technology, it is crucial to develop new materials with properties that outperform “old” materials. Thus, humanity has been preoccupied since ancient times with improving its standard of living, searching for new materials that can help and facilitate their day-to-day tasks. In recent years, the activities of AMs have become a common focus of research in many fields, and, in regard to biomedical applications, they are one of the most explored subjects. Figure 1 shows how the relative importance of materials in the economy has changed with time. The width of each band depicts, as a percentage, the contribution of each family of AM (metals, polymers, composites, ceramics, and glasses) to the economy of the corresponding age and, in some cases, the present day.

In this Special Issue, entitled “Advanced Materials for Biomedical Applications”, the goal is to bring together advances in material development to be applied (but not limited to) to areas such as wound healing, cancer treatment (and other illnesses, e.g., diabetes), biosensing, catheters, hygiene products, scaffolds, bone regeneration, and drug delivery. AMs are considered to include, but are not limited to, metallic and oxidic nanostructures, ceramics, polymers, supramolecular assemblies (e.g., microemulsions, liposomes), and alloys.

We sincerely hope that the contributions to this Special Issue will improve patients' quality of life and provide a route to saving lives worldwide. Additionally, our goal is to encourage new research in the promising area of “Advanced Materials for Biomedical Applications”.

## Figures and Tables

**Figure 1:**
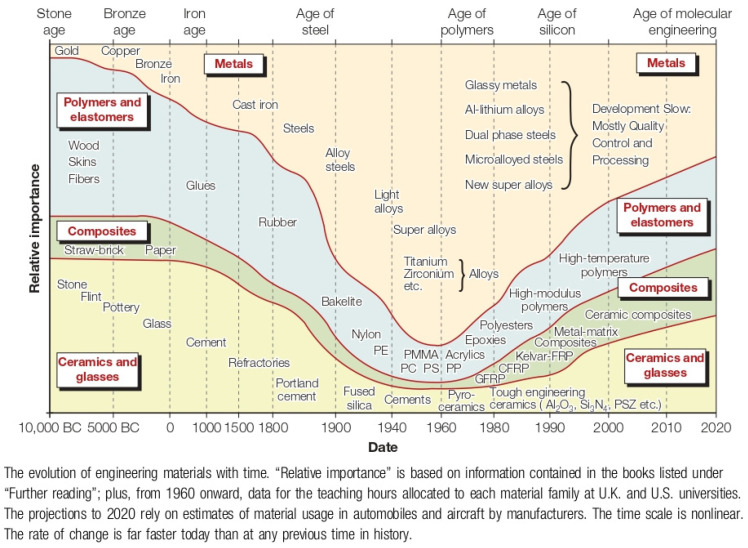
Historical perspective of materials according to their relative importance [2]. Reprinted with permission from Ashby, M. F. (2011). Introduction. Materials Selection in Mechanical Design, 1–13. doi:10.1016/b978-1-85617-663-7.00001-1.
